# Early comparison of one-year outcomes after aortic mechanical vs. biological valve replacement in Chinese patients with small aortic annulus

**DOI:** 10.1186/s13019-025-03614-4

**Published:** 2025-12-05

**Authors:** Yu Sun, Yang Gao, Wen Xiao, Zimo Wang, Yonglei Liu, Xiao Wang, Laichun Song

**Affiliations:** 1 Department of Cardiovascular Surgery, Wuhan Asia Heart Hospital, Wuhan University of Science and Technology, No. 753 Jinghan Avenue, Jianghan District, Wuhan, 430022 Hubei China; 2 Department of Anaesthesiology, Wuhan Asia Heart Hospital, Wuhan University of Science and Technology, Wuhan, Hubei China

**Keywords:** Small aortic valve annulus, Aortic valve replacement, Bioprosthetic valve, Mechanical valve

## Abstract

**Background:**

This study compares short-term clinical and hemodynamic outcomes of AVR using mechanical versus bovine pericardial valves in patients with a small aortic annulus (≤ 21 mm) and evaluates the feasibility and safety of AVR in this population.

**Methods:**

Between November 2020 and November 2022, 142 patients with a small aortic valve annulus underwent AVR at Wuhan Asia Heart Hospital, with 73 patients in mechanical group and 69 in biological group. All patients met the surgical criteria for AVR. Baseline data, historical diseases, preoperative and follow-up echocardiographic results, and surgical-related data were shown in this study.

**Results:**

All patients successfully underwent surgery. In the mechanical valve group, four perioperative deaths occurred due to multiple organ failure, respiratory and cardiac arrest and circulatory failure. After one-year follow-up, hemodynamic parameters improved in both groups compared with preoperative values. Although the mechanical valve group demonstrated lower peak pressure gradients and maximum velocity compared with the bioprosthetic valve group in the follow-up period (*P* < 0.05), the bioprosthetic valve group exhibited shorter Intensive Care Unit (ICU) stay(3.6 ± 2.5d vs. 5.3 ± 7.9d, *P* < 0.001 ) and cardiopulmonary bypass (CPB) times (178.1 ± 89.0 min vs. 192.4 ± 124.3 min, *P* = 0.032), and fewer postoperative complications at the one-year follow-up (4.3% vs. 14.9%, *P* = 0.026). No cerebrovascular events, valve dysfunction, or structural valve degeneration were observed in either group during the follow-up period.

**Conclusions:**

Both mechanical and bioprosthetic valves showed excellent short-term outcomes in small aortic annulus patients. Bioprosthetic valves were comparable to mechanical valves, with potential benefits in reducing ICU stay, CPB time, and complications. For elderly patients, bioprosthetic valves are a safe and effective alternative in small aortic annulus AVR.

## Introduction

Selecting the appropriate prosthesis for aortic valve replacement (AVR) in patients with a small aortic annulus remains a significant surgical challenge [1]. AVR with small prosthetic valves, though widely performed, carries the inherent risk of prosthesis–patient mismatch (PPM), typically defined as an indexed effective orifice area (EOAi) less than 0.85 cm²/m² [2], which may lead to suboptimal haemodynamics and increased postoperative outflow gradients. These elevated gradients may impair left ventricular mass regression and contribute to adverse outcomes, including stroke, ischemic events, and mortality. Patients with aortic stenosis (AS) and a small aortic annulus or narrower aortic roots face additional challenges, as these anatomical limitations complicate valve implantation and may necessitate annular enlargement [3, 4]. While annular enlargement can facilitate the insertion of larger prosthetic valves, it carries increased surgical risk [5]. Similarly, homografts can offer lower postoperative pressure gradients, but are technically demanding, involve prolonged ischemic times, and may be less feasible in low-resource settings [6, 7]. The pathophysiological mechanism by which PPM leads to adverse outcomes involves chronic pressure overload on the left ventricle, limited regression of left ventricular hypertrophy, and persistent diastolic dysfunction. These factors underscore the importance of evaluating alternative prosthetic options for small aortic annulus patients [7, 8].

The Cingular pericardial valve, a stented bovine pericardial tissue valve, has demonstrated promising haemodynamic performance in small annuli. Evidence from prior studies suggests its superiority over other similarly sized biological valves [9–11]. In comparison, bileaflet mechanical valves, such as the Medtronic-AP360™, remain a commonly used alternative due to their durability and established clinical efficacy [12, 13].

This current study aimed to compare the early-term clinical and haemodynamic results of biological valves (Cingular Valve™) and mechanical valves (Medtronic-AP360™) in patients with small prosthetic valves. By evaluating the safety, feasibility, and short-term performance of these two prostheses, this study seeks to provide insights into optimizing AVR outcomes for this challenging patient population.

## Methods and materials

### Ethics statement and consent to participate

The Research Ethics Committee of Wuhan Asia Heart Hospital, Wuhan University of Science and Technology, Wuhan, Hubei, China, provided ethical permission for this study. Informed consent was obtained from all individual participants included in the study. All data were anonymized to ensure participant confidentiality.

## Study design

This retrospective study included 142 patients with aortic valve disease who underwent aortic valve replacement (AVR) at Wuhan Asia Heart Hospital between November 2020 and August 2022. Patients were eligible for inclusion if they were diagnosed with aortic valve disease (degenerative, congenital, or rheumatic), including aortic stenosis with or without regurgitation, and had a small aortic annulus (≤ 21 mm). Patients were excluded if they had severe comorbidities requiring complex or concomitant surgery (e.g., coronary artery bypass grafting, aortic root or ascending aortic replacement), severe systemic diseases (e.g., end-stage renal failure), or incomplete follow-up data. All included patients underwent isolated aortic valve replacement. The study aimed to evaluate the short-term clinical and haemodynamic outcomes of AVR using biological valves (Cingular Valve™, sizes 19 mm and 21 mm) versus mechanical valves (Medtronic-AP360™, sizes 18 mm and 20 mm). The Cingular Valve™ (Cingular Biotech, Shanghai, China), a stented bovine pericardial valve designed for small aortic annuli, is known for its superior haemodynamic performance. The 142 patients were divided into two groups, with 73 patients in the mechanical group and 69 in biological group. The choice of valve type was based on the latest clinical guidelines, patient age, long-term anticoagulation suitability, patient preference, and surgeon recommendations.

## Surgical procedure

All patients underwent surgery via median sternotomy. No minimally invasive approaches, such as right anterior thoracotomy, were used. After systemic heparinization, cardiopulmonary bypass (CPB) was established through ascending aortic and bicaval cannulation in all cases. Myocardial arrest was achieved using cold blood cardioplegia. A transverse aortotomy was performed approximately 1 cm above the sinotubular junction.

The native aortic valve was excised, and thorough annular decalcification was performed. Valve size was selected based on annular measurements and body surface area. In both groups, either a mechanical or bioprosthetic valve (19–21 mm) was implanted using interrupted pledgeted sutures. No annular enlargement was performed. After de-airing, the aortotomy was closed, and the cross-clamp was removed. All patients were weaned from CPB uneventfully. Intraoperative TEE confirmed valve function and absence of paravalvular leak. No concomitant procedures were performed.

## Study and points

The mean follow-up duration was 319.0 ± 307.5 days, with a mean duration of 308.6 ± 304.0 days in the mechanical group and a mean duration of 346.3 ± 310.9 days for the biological group. This study included baseline data, surgery-related data, and one-year follow-up data. The following data were collected from patients at baseline: age, sex, body surface area (BSA), cardiac function classification, valve size, and disease history (including hypertension, diabetes mellitus, and coronary heart disease). No patients had a history of cardiac surgery. The following surgery-related data were collected: cardiopulmonary bypass (CBP) time, intensive care unit (ICU) stay, in-hospital mortality, and postoperative complications. The following echocardiographic follow-up data were collected: the left ventricular ejection fraction (LVEF), left atrial (LA) diameter, peak pressure gradient (PG), Valve Maximum Velocity and left ventricular end-diastolic diameter (LVEDD).

## Data management and statistics

All variables in this study were statistically analysed according to variable type. Categorical variables are presented as frequencies with percentages and were compared using the chi-square test or Fisher’s exact test. Continuous variables are expressed as mean ± standard deviation and ***IQR*** and were compared using the ***t*** test and Mann-Whitney U test. All the statistical analyses were performed using Statistical Package for Social Science version 26 (SPSS Inc., IBM Corp., Chicago, Illinois), a ***P*** value < 0.05 was considered to indicate statistical significance.

## Results

### Baseline characteristics in two groups

This study included 142 patients who underwent AVR from November 2020 to August 2022, with 73 patients in mechanical group and 69 in biological group. The mean age was 61.6 ± 6.8 years in total, with mean ages of 58.9 ± 6.3 years for the mechanical group and 64.3 ± 6.3 years for the biological group. Among patients with mechanical valves, 28.8% had hypertension, 5.5% had diabetes mellitus, and 11.0% had coronary heart disease, while 58.0% had hypertension, 13.0% had diabetes mellitus, and 46.4% had coronary heart disease in biological group. No statistically significant differences were found between the two groups in terms of age, sex, BSA, aortic stenosis, aortic insufficiency, valve size, diabetes mellitus. The data were presented in Table 1.

**Table 1:** Baseline characteristics in two groups

Label	Mechanical group (N = 73)	Biological group (N = 69)	*P*- value
Age, year	58.9 ± 6.3	64.3 ± 6.3	0.640
**Sex**,** n (%)**			
Male	6 (8.2)	9 (13.0)	0.587
Female	67 (91.8)	60 (87.0)	
Follow-up Days, days	308.6 ± 304.0	346.3 ± 310.9	0.733
Min-Max, days	(4.0,1026.0)	(3.0,955.0)	
**Height**,** cm**	154.2(***IQR***:149.0,158.0)	156.3(***IQR*** :152.0,160.5)	0.053
**Weight**,** Kg**	57.0(***IQR*** :51.0,62.5)	58.4(***IQR***:51.0,65.5)	0.377
**BSA**,** m**^**2**^	1.6(***IQR*** :1.5,1.7)	1.7(***IQR*** :1.5,1.8)	0.189
**LVEF%**			
≤ 50%	22 (30.1)	21 (30.4)	0.856
> 50%	51 (69.9)	48 (69.6)	
**Aortic Stenosis**,** n (%)**	46 (63.0)	52 (75.4)	0.379
**Aortic Insufficiency**,** n (%)**	62 (84.9)	63 (91.3)	1.000
**Valve Size**,** n (%)**			
19 mm	14 (19.2)	24 (34.8)	0.089
21 mm	59 (80.8)	45 (65.2)	
**NYHA**,** n (%)**			
Ⅲ+Ⅳ	40(54.8)	34(49.3)	0.666
**Past History**,** n (%)**			
Hypertension	21 (28.8)	40 (58.0)	**0.002**
Diabetes Mellitus	4 (5.5)	9 (13.0)	0.244
Coronary Artery Disease	8 (11.0)	32 (46.4)	**< 0.001**

For statistical variables (e.g., IQR), bold italics are applied consistently throughout the tables to highlight statistical terms

##  Surgical and 1-year follow-up data outcomes

The ICU length of stay was shorter in the biological group compared to the mechanical group (3.6 ± 2.5 days vs. 5.3 ± 7.9 days, *P* < 0.001). Statistically significant differences were also observed in CPB time (178.1 ± 89.0 min vs. 192.4 ± 124.3 min, *P* = 0.032). Neither group had patients with PPM or an effective orifice area index (EOAi) greater than 0.85 cm²/m². The mechanical group experienced a higher rate of postoperative complications than the biological group (*P* = 0.026), and the in-hospital mortality rate was 5.4% in the mechanical group, compared to 0% in the biological group (*P* < 0.044). There were 11 cases of postoperative complications in the mechanical valve group and 3 cases of postoperative complications in the biological valve group (*P* = 0.026) during the follow-up period. In addition, 5 cases of paravalvular leak were observed, all graded as mild to moderate on echocardiography, with no immediate reintervention required. Three cases of mild valvular regurgitation, 1 case of endocarditis, and 2 cases of Atrial Fibrillation (AF) occurred in the mechanical valve replacement group; the atrial fibrillation were new-onset atrial fibrillation managed medically without permanent pacemaker implantation. In the biological valve replacement group, 2 cases of mild paravalvular leak and 1 case of arrhythmia (paroxysmal atrial fibrillation relieved after rate control and rhythm stabilization) were detected and effectively relieved after intervention treatment. These data were shown in Table 2. No reintervention or reoperation events were recorded during the follow-up period.

**Table 2:** Postoperative and Follow-up data between two groups

Label	Mechanical group (N = 73)	Biological group (N = 69)	*P*- value
In-hospital mortality, n (%)	4(5.4%)	0(0.0%)	**0.044**
CPB time, min	192.4 ± 124.3	178.1 ± 89.0	**0.032**
ICU time, day	5.3 ± 7.9	3.6 ± 2.5	**< 0.001**
**1-Year Total Postoperative Complications**,** n (%)**	**11(14.9)**	**3(4.3)**	**0.026**
Perivalvular Leak (mild–moderate)	5(6.8)	2(2.9)	
Valve Regurgitation (mild)	3(4.1)	0(0.0)	
Endocarditis	1(1.4)	0(0.0)	
Atrial fibrillation (AF)	2(2.7)	1(1.4)	
**1-Year EOA**,** cm²**			
19 mm	1.56	1.62	0.639
20 mm	1.82	1.77	**0.036**
**1-Year EOAi**	1.6 (***IQR*** :1.4,1.7)	1.0 (***IQR*** :0.9,1.1)	**< 0.001**
**1-Year PPM**,** n (%)**			
> 0.85cm^2^/m^2^	70(95.9)	69(100.0)	**-**
≤ 0.85cm^2^/m^2^	0	0	
≤ 0.65cm^2^/m^2^	0	0	
Missing	3 (4.1)	0	

For statistical variables (e.g., IQR), bold italics are applied consistently throughout the tables to highlight statistical terms

### Echocardiographic data before and after operation

There were no significant differences in LVEF%, LA, and LVEDD between the two groups after operation (*P* > 0.05). The peak PG was 50.3 ± 9.8 mmHg before the operation and 41.4 ± 10.2 mmHg at the one-year follow-up in the mechanical group while 60.9 ± 45.4 mmHg before the operation and 42.0 ± 10.8 mmHg in the one-year follow-up period in the biological group. The peak PG (*P* = 0.037) and valve maximum velocity (2.3 ± 0.6 m/s vs. 2.4 ± 0.4 m/s, *P* = 0.049) was lower in the mechanical group at the one-year follow-up. Details were shown in the Table 3.

**Table 3:** Echocardiographic data before and after operation between groups (Follow-Up data)

Label	Mechanical group (N = 73)	Biological group (N = 69)	*P*- value
LVEF, %			
Before	53.8 ± 4.5	53.3 ± 5.2	0.523
After	53.5 ± 3.7	53.4 ± 4.1	0.789
**LA**,** mm**			
Before	50.3 ± 9.8	47.7 ± 12.4	0.155
After	41.4 ± 10.2	42.0 ± 10.8	0.713
**Peak PG**,** mmHg**			
Before	51.6 ± 34.2	60.9 ± 45.4	**0.008**
After	22.6 ± 5.3	24.7 ± 6.0	**0.037**
**Valve Maximum Velocity**,** m/s**			
Before	3.4 ± 1.2	4.0 ± 1.0	**< 0.001**
After	2.3 ± 0.6	2.4 ± 0.4	**0.049**
**LVEDD**,** mm**			
Before	48.1 ± 7.7	50.2 ± 5.9	0.064
After	45.1 ± 3.5	45.9 ± 3.8	0.188

## Discussion

In an aging society, the prevalence of heart valve disease is steadily increasing [14]. This trend is particularly pronounced in East Asia, where smaller body frames and a higher prevalence of small aortic annuli are observed [15]. Small aortic valve disease poses unique challenges for aortic valve replacement (AVR), as these patients are more likely to experience poor postoperative outcomes [16], including higher mortality rates, prosthesis-patient mismatch (PPM), and an increased risk of complications such as myocardial hypertrophy and congestive heart failure [17, 18]. Addressing these challenges requires tailored surgical strategies to optimize outcomes and minimize complications [19]. Surgical approaches to mitigate PPM [20], such as annular enlargement, Ross procedures, and stentless bioprostheses, are associated with additional risks and technical challenges [19]. These methods may involve prolonged surgical times, significant learning curves, higher costs, or limited availability, making them less favorable in certain clinical settings [21–23].

In this study, we compared the Medtronic AP360 20-mm mechanical valve with a 21-mm bioprosthetic valve due to the inherent design differences between mechanical and bioprosthetic valves. The 20-mm mechanical valve lacks a direct counterpart in the 21-mm size category, and its effective orifice area (EOA) is often clinically equivalent to or greater than that of a 21-mm bioprosthetic valve. This reflects real-world clinical decision-making, where such comparisons are routinely made based on anatomical and functional considerations. PPM, a key issue in small aortic valve annuli, occurs when the prosthetic valve’s effective orifice area (EOA) is too small for the patient’s body surface area. This leads to elevated left ventricular afterload, increased pressure gradients, and left ventricular outflow tract obstruction, ultimately compromising the patient’s recovery [17, 18]. Surgical approaches to mitigate PPM, such as annular enlargement, Ross procedures, or stentless bioprostheses, can improve EOA but are often associated with higher technical complexity, prolonged operative times, steep learning curves, and greater risks of complications [19, 20]. For instance, the Ross procedure, while effective for younger patients, is rarely used in adults due to its demanding nature and associated risks. Stentless bioprostheses provide excellent hemodynamics but are costly and less available in many regions, including China. These limitations highlight the need for less invasive, safer, and more cost-effective alternatives for patients with small annuli.

In China, mechanical valves are the most commonly chosen option for AVR due to their affordability and robust hemodynamic performance [24, 25]. However, mechanical valves require lifelong anticoagulation therapy, which presents a significant burden for patients, especially those with contraindications to anticoagulants or poor adherence to medication regimens. In contrast, bioprosthetic valves, despite concerns about durability, are increasingly used in AVR because they improve quality of life, eliminate the need for anticoagulation, and simplify postoperative management [26, 27]. The Cingular Valve™, a stented bovine pericardial valve specifically designed for small aortic annuli in Asian populations, represents a significant advancement in this field. Compared to similarly sized valves, the Cingular Valve™ offers a larger EOA, better hemodynamic performance, and reduced transvalvular pressure gradients [9–11]. These features address the anatomical challenges posed by small annuli and help mitigate PPM. Furthermore, bioprosthetic valves are compatible with transcatheter aortic valve replacement (TAVR), enabling minimally invasive interventions in the future and potentially avoiding repeat open-heart surgeries. This compatibility enhances long-term patient management, especially in elderly patients or those with multiple comorbidities.

Among the patients included in the study, those receiving bioprosthetic valves showed comparable short-term hemodynamic performance to those receiving mechanical valves. The bioprosthetic group experienced significantly fewer postoperative complications, shorter ICU stays and reduced cardiopulmonary bypass times, it may because the lower NYHAⅢ and Ⅳ at the baseline. Additionally, the recovery of left ventricular ejection fraction (LVEF) was significantly better in the bioprosthetic group, reflecting improved myocardial performance. While the peak pressure gradient and valve maximum velocity was higher in the bioprosthetic group after operation, this difference can be attributed to factors such as the smaller valve sizes used in this group (34.8% of patients in the bioprosthetic group received 19-mm valves compared to 19.2% in the mechanical group). Additionally, mechanical valves of a given nominal size typically provide better EOA compared to bioprosthetic valves, which explains the observed differences [28]. Interestingly, the in-hospital mortality rate was significantly higher in the mechanical valve group, with four perioperative deaths attributed to complications such as multiple organ failure and multidrug-resistant infections. These findings differ from previous studies that reported higher survival rates in mechanical valve recipients [29, 30]. This discrepancy may be explained by socioeconomic factors, as patients with lower income often choose mechanical valves due to their lower cost but may face limited access to postoperative follow-up, inadequate nutritional support, and a higher burden of baseline comorbidities [31–33]. In addition, the small sample size may amplify the statistical impact of a few adverse events. These factors underscore the importance of considering patient-specific circumstances, including economic status, when selecting prosthetic valves. The results of this study suggest that bioprosthetic valves are a safe and effective alternative for AVR in patients with small aortic annuli. Their comparable hemodynamic performance, coupled with fewer complications and better recovery, makes them a viable option for elderly patients or those with contraindications to anticoagulation therapy. Additionally, their compatibility with TAVR enhances their long-term utility, addressing the growing need for minimally invasive solutions in an aging population.

This study underscores the importance of selecting the most suitable prosthesis for AVR in patients with small aortic annuli, a population with unique clinical challenges, particularly in East Asia. By providing valuable haemodynamic evidence and demonstrating the comparable efficacy of biological valves, particularly the Cingular Valve™, this study highlights a promising alternative for small aortic annulus AVR. These findings not only support the safety and feasibility of biological valves but also address the increasing demand for better quality-of-life options in an aging society. This research contributes to optimizing clinical decision-making and may guide future surgical practices for this challenging subgroup.

### Limitation

The main limitation of this study is its non-randomized retrospective design, which may introduce selection bias and limit the generalizability of the findings. Significant baseline differences between groups, such as historical diseases, could still affect the results. The relatively small sample size also reflects the declining number of isolated small-annulus SAVR cases in the TAVR era. Additionally, the short follow-up period limits the evaluation of long-term outcomes. Immediate postoperative echocardiographic data were not uniformly available, limiting the assessment of early valve performance. Mean pressure gradients were unavailable in many reports; thus, only peak gradients were analyzed. Future studies with larger sample sizes, longer follow-up, and prospective randomized designs are needed to validate these findings.

## Conclusions

This study demonstrates that aortic valve replacement with a small bioprosthetic valve is a safe and effective option for patients with a small aortic valve annulus (≤ 21 mm). The short-term hemodynamic outcomes of bioprosthetic valves are comparable to those of mechanical valves, with additional benefits such as reduced ICU stay, shorter CPB times, and fewer postoperative complications. These findings suggest that bioprosthetic valves are a viable and advantageous alternative, particularly for elderly patients, in this challenging patient population.

## Data Availability

Data is provided within the manuscript files.

## Abbreviations

SAVR: Surgical aortic valve replacement
AI: Aortic insufficiency
AS: Aortic stenosis
AR: Aortic regurgitation
AVR: Aortic valve replacement
AF: Atrial fibrillation
TTE: Transthoracic echocardiogram
LVEF: Left ventricular ejection fraction
BSA: Body surface area
EOAi: Indexed effective orifice area
PPM: Prosthesis patient mismatch
NYHA: New York heart association
PG: Pressure gradient
LA: Left atrium
LVEDD: Left ventricular end-diastolic diameter
IQR: Interquartile range
SD: Standard deviation
HR: Hazard ratio
CI: Confidence interval
CPB: Cardiopulmonary bypass
ICU: Intensive care unit
BSA: Body surface area
